# Testosterone and depressive symptoms during the late menopause transition

**DOI:** 10.1186/s13293-021-00388-x

**Published:** 2021-07-30

**Authors:** Bethany Sander, Amira Muftah, Laurie Sykes Tottenham, Julia A. Grummisch, Jennifer L. Gordon

**Affiliations:** Department of Psychology, University of Regina, Regina, Saskatchewan Canada

**Keywords:** Testosterone, Estradiol, Testosterone-to-estradiol ratio, Depressive symptoms, Vasomotor symptoms, Sleep, Menopause transition

## Abstract

**Background:**

The menopause transition is associated with an increased risk of depression. While the mechanisms behind this increased risk are not well understood, the changing perimenopausal hormonal environment has been hypothesized to play a role. The current study examined the potential influence of testosterone and the ratio of testosterone to estradiol as a potential contributor to depressed mood in the menopause transition.

**Methods:**

Fifty non-depressed perimenopausal women ages 45–55 were recruited for this study. Once every 3 weeks, for a total of four times, the women completed the Centre for Epidemiological Studies-Depression (CES-D) scale for the measurement of depressive symptoms and provided a first-morning urine sample for the measurement of urinary testosterone as well as estrone-3-glucuronide (E1G), a urinary metabolite of estradiol. The week-to-week and mean effects of testosterone, E1G, and the testosterone/E1G ratio on CES-D score were examined. Self-reported sleep quality and vasomotor symptoms were also assessed at each of the four time points.

**Results:**

Testosterone levels rose with increasing months since last menstrual period associated with testosterone levels (*β*(SE) = 175.3(63.2), *p* = .006), though this effect was moderated by body mass index (*p* for the interaction = .001) such that overweight women showed a less pronounced increase over time. Past and current smokers also had higher testosterone levels compared to never smokers. Week-to-week testosterone/E1G ratio was positively associated with CES-D score (*β*(SE) = 1.57(0.76), *p* = .041) but not sleep quality or vasomotor symptoms (*p*s > .05). Mean testosterone/E1G ratio was also positively associated with vasomotor symptom bother (*β*(SE) = 0.14(0.06), *p* = .018) and poorer sleep quality (*β*(SE) = − 0.34(0.09), *p* = .0001).

**Conclusion:**

These results suggest that, within the context of the menopause transition, times that are characterized by a higher testosterone-to-estradiol ratio may be associated with higher depressive symptoms. Perimenopausal women with a higher average ratio of testosterone relative to estradiol may also experience more sleep difficulties and vasomotor symptom bother.

The menopause transition, or perimenopause, represents the progression from regular ovulatory menstrual cycles to the complete cessation of menstruation. A large body of research suggests that the menopause transition is a time of increased risk for depressive symptoms [1]. Although estradiol withdrawal [2] and increased estradiol fluctuation [3] have been implicated in the development of these symptoms, the current study aimed to examine the importance of a second, less studied, potential contributor: that of androgen excess.

Studies that have examined levels of androgens, such as testosterone, in relation to mood in the menopause transition have obtained inconsistent findings, with some supporting a positive relationship between testosterone and depressive symptoms [4, 5] and others observing either an inverse relationship [6] or no relationship at all between testosterone and mood [7]. There is also a lack of clarity about the trajectory of testosterone levels throughout the menopause transition, with one longitudinal study reporting stable testosterone levels across the menopausal transition [4, 8], another reporting a decline in testosterone [9], and still another reporting a rise in testosterone levels [5].

The current study aimed to (1) help clarify the trajectory of testosterone levels across the menopause transition and (2) examine testosterone levels in relation to mood and other perimenopausal symptoms. With regard to the first objective, we aimed to extend the current literature by examining potential moderators of the progression of testosterone levels with advancing reproductive age as the existence of such moderators might help explain observed inconsistencies in the current research. With respect to the second objective, we aimed to investigate not only testosterone levels, but also the testosterone-to-estrogen ratio, as a measure of overall androgenicity of the hormonal environment. Indeed, some research has pointed to the testosterone-to-estradiol ratio as being a better predictor of clinical outcomes, such as incident metabolic syndrome [10] and vasomotor symptoms [11], over and above levels of either hormone alone. Thus, investigating the mood effects of testosterone while also accounting for estrogen levels could potentially help clarify testosterone’s mood effects in the menopause transition.

## Methods

### Study population

The data reported herein are derived from a sub-study of the Fluctuating Estrogen and Menopausal Mood (FEMM) Study [12], whose primary aim was to examine individual differences in mood sensitivity to estradiol fluctuation. Women recruited from the community were between 45 and 55 years and perimenopausal according to the Stages of Reproductive Aging Workshop (STRAW+10) criteria (early perimenopause, defined as a menstrual cycle length variability from usual of 7+ days; late perimenopause, defined as at least 2 skipped cycles and an interval of amenorrhea of at least 60 days but less than 12 months) [13]. They also had to report having regular menstrual cycles throughout most of their reproductive lifespan — thus, women with polycystic ovarian syndrome and congenital adrenal hyperplasia were excluded from this study. Additional exclusion criteria included the following: current psychiatric diagnosis of major depressive disorder (MDD), bipolar disorder, any other psychiatric diagnosis rated “severe” based on DSM-5 criteria, current use of medications affecting mood or ovarian hormonal levels, and current pregnancy or nursing.

### Procedure

Following an in-person enrollment session during which eligibility was confirmed and baseline measures were completed using an online survey, participants completed 12 weeks of biosample collection, and vasomotor symptom (VMS) monitoring. Specifically, on the same day each week (chosen by the participant), starting at 6 pm, participants were prompted via email or text to begin recording their VMS using a paper-based 24-h hot flash diary. The following morning, participants were asked to tally the number of mild, moderate, and severe hot flashes they had experienced and to enter them in an online survey emailed to them. In that same survey, participants completed the Center for Epidemiologic Studies-Depression Scale [14] and rated the quality of their sleep on the previous night using a 5-point scale.

The morning following each weekly mood assessment, participants collected 4 ml of their first-morning voided urine for the measurement of both estrone-3-glucuronide (E1G) and pregnanediol glucuronide (Pdg), which are urinary metabolites of estradiol and progesterone, respectively. At the end of study phase 1, samples were mailed to the university via a same-day courier service and frozen at − 40 °C until assayed. These metabolites have been shown to correlate very highly (*r*s = 0.93–0.97) with serum levels of estradiol and progesterone measured 1 day prior to urine collection [15]. In other words, first-morning urine levels of E1G and PdG reflect an integrated measure of the overall hormone levels from the previous day. For this reason, urine collection occurred the morning following the completion of the CES-D. A recent study has validated the measurement of these metabolites in perimenopausal women [16].

The primary objective of the FEMM study was to examine individual sensitivity to estradiol as a longitudinal predictor of perimenopausal depressive symptoms. The results of these analyses are published elsewhere [12]. For the current sub-study, the urine samples of the last 50 participants were assayed for urinary testosterone at weeks 3, 6, 9, and 12. Urinary testosterone is recognized as a valid measure of testosterone production [17] that has been shown to correlate highly with serum testosterone levels following administration of exogenous testosterone in both young women (*r* = 0.69) [18] and male athletes (*r* = 0.56) [19]. The Research Ethics Board of the University of Regina approved the study protocol. All participants provided written informed consent and were compensated up to $250 for participating in full compliance.

### Baseline measures

The Structured Clinical Interview for DSM-5 (SCID-5) was administered at baseline to obtain information about past and current psychiatric disorders [20]. The Center for Epidemiological Studies-Depression Scale [14] and State-Trait Anxiety Inventory-Form Y2 [21] were administered to assess depressive symptoms and trait anxiety, respectively. The Pittsburgh Sleep Quality Index [22] was used to assess sleep quality. Additionally, age, years of education, race, smoking status, number of past diagnoses of major depressive disorder, months since last menstrual period, body mass index, and STRAW stage [13] were assessed at baseline.

### Repeated measures

#### Center for Epidemiological Studies-Depression Scale (CES-D)

The CES-D is a 20-item self-report measure that assesses the frequency of depressive symptoms during the previous week on a 4-point scale of 0 (rarely or never) to 3 (most or all of the time) [14]. A score of 16 or higher is generally used to identify potential clinical depression [23].

#### Subjective sleep quality

Sleep quality was assessed using a single question extracted from the Pittsburgh Sleep Quality Index [22] in which participants rated the quality of their sleep on a scale from 1 to 5 (1 = very poor; 5 = very good).

#### Self-reported vasomotor symptoms (VMS)

Items 19 and 20 from the Greene Climacteric Scale [24] were also administered weekly, asking participants to rate on a scale from 0 to 3 how bothered they were by their current hot flashes and night sweats, respectively. The mean of these items provided an indicator of VMS bother. Using a booklet, participants also wrote down each hot flash and night sweat experienced over a 24-h period and rated the severity of each — mild, moderate, or severe. Severity definitions provided followed FDA guidelines for defining hot flash severity [25]. The total number of VMS was calculated.

### Enzyme immunoassays

Urinary testosterone was assayed using a High Sensitivity ELISA kit (Enzo Life Sciences, Farmingdale, NY). The respective intraassay and interassay coefficients of variation were 4.59% and 8.36%. Estrone-3-glucuronide (E1G), a urinary metabolite of estradiol, was assayed using an enzyme immunoassay (Arbor Assays, Ann Arbor, MI). The intraassay coefficient of variation was 5.1% and the interassay coefficient of variation was 14.8%. To account for differences in urine concentration, E1G and testosterone levels were adjusted for specific gravity using the formula recommended by O’Connor and colleagues [26].

### Statistical analyses

PROC MIXED in SAS 9.4 was used to examine the effect of both weekly levels and mean levels of E1G, testosterone, and the T/E1G ratio on depressive symptoms, sleep quality rating, VMS number, and VMS bother. When examining mean levels of E1G, sleep, and VMS, all 12 available weekly outcomes were included; for models examining the week-to-week effects of hormones, only the four repeated measures that were paired with the 4 weeks of testosterone measurement were included. Models were fitted using a restricted maximum likelihood (REML) estimation method. A first-order autoregressive covariance structure for within-person error was applied and the Kenward-Rogers correction was used to calculate the appropriate degrees of freedom. A repeated statement specified that weeks were nested within subjects.

## Power calculations

Power analyses were carried out as sensitivity analyses as the sample size chosen was primarily based on funds available to assay testosterone levels. With regard to the week-to-week effects of hormones, for each psychological outcome, the observed intraclass correlation in the sample was used to estimate the smallest detectible effect size *f*^2^ [27]. For weekly depressive mood, the smallest detectible effect size was calculated to be *f*^2^ =.11; for VMS number, it was *f*^2^ =.066; for VMS bother, it was *f*^2^ =.099; for sleep quality, it was *f*^2^ =.075. Given the following local effect size conventions for multilevel regression coefficients (or functional sets of coefficients): .02 = small effect, .15 = medium effect, and .35 = large effect [28], this study was powered to detect conventionally small-to-medium effects of week-to-week hormones. Since the number of repeated measures was greater for models estimating the effects of mean hormone level effects, these were powered to detect even smaller effects.

## Results

### Participant characteristics

Table 1 describes participant characteristics at baseline and across the 12-week study. As a result of our community-based recruitment, most participants were Caucasian and late-perimenopausal at the time of enrollment. Most participants were either never smokers or past smokers; on average, participants were overweight.

**Table 1:** Participant characteristics

Baseline characteristics	
Age, mean (SD)	49.8 (2.6)
Years of education, mean (SD)	13.2 (5.8)
Education level, (%)	
High school diploma	22%
Some university	33%
Undergraduate degree	29%
Graduate degree	16%
Family income, mean (SD), 10K	
< $70,000	17%
$70,000–89,999	19%
$90,000–112,999	15%
≥ $113,000	43%
No response	6%
Ethnicity, (%)	
Caucasian	86%
Black	2%
Hispanic	4%
Others	8%
Months since LMP, mean (SD)	2.2 (1.7)
Baseline STRAW status (%)	
Early perimenopause	34%
Late perimenopause	66%
STRAW status at the end of phase 1 (%)	
Early perimenopause	8%
Late perimenopause	84%
Early postmenopause	5%
Body mass index (BMI; kg/m^2^)	29.4 (6.5)
Overweight (25 ≤ BMI < 29.9 kg/m^2^)	38%
Obese (BMI ≥ 30 kg/m^2^)	36%
Smoking status (%)	
Never smokers	57%
Past smokers	34%
Current smokers	10%
History of major depression (%)	38%
**Weekly variables**	
CES-D score, mean (SD)	12.0 (8.0)
Number of hot flashes, mean (SD)	
Mild	1.9 (1.1)
Moderate	1.7 (1.0)
Severe	1.4 (1.0)
Sleep quality score, mean (SD)	2.3 (0.9)
E1G, mean (SD), pg/ml	39,624 (19,809)
Testosterone, mean (SD), pg/ml	2113 (1295)
Testosterone-to-E1G ratio, mean (SD)	0.068 (0.055)

*Abbreviations*: *LMP* last menstrual period, *MDD* major depressive disorder

### Baseline predictors of testosterone (T), E1G, and the T/E1G ratio

**Fig. 1:**
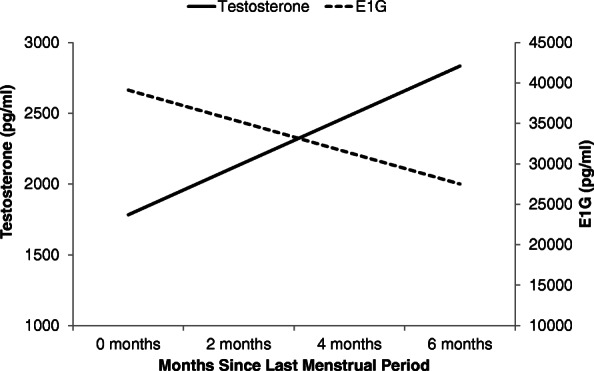
Model-based estimates of testosterone and E1G levels by number of months since last menstrual period

Race (*p*s > .158), age (*p*s > .364), family income (*p*s > .500), years of education (*p*s > .109), and history of major depressive disorder (*p*s > .080) were not associated with E1G, T, or the T/E1G ratio. E1G levels were weakly positively associated with testosterone levels (*β*(SE) = 0.01(0.00), *p* = .039). Number of months since a woman’s last menstrual period (LMP) was positively associated with testosterone levels (*β*(SE) = 175.3(63.2), *p* = .006) and negatively associated with E1G levels (*β*(SE) = − 1936.4(735.0), *p* = .009) (Fig. 1), resulting in a positive association between number of months since a woman’s LMP and the T/E1G ratio (*β*(SE) = 0.01(0.00), *p* < .001).

**Fig. 2:**
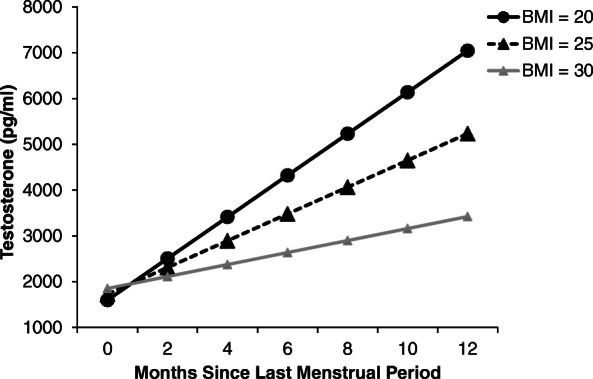
Model-based estimates of testosterone and months since last menstrual period by body mass index

**Fig. 3:**
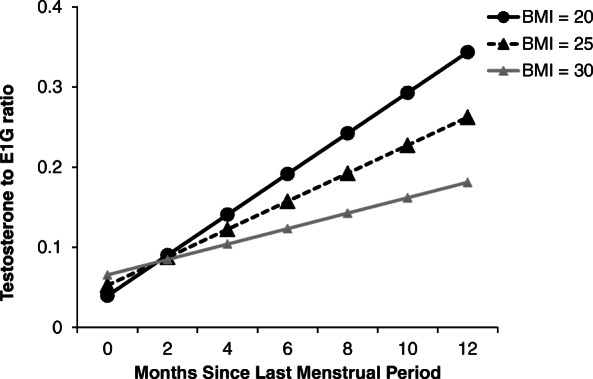
Model-based estimates of the testosterone-to-E1G ratio and months since last menstrual period by body mass index

BMI was negatively associated with both testosterone (*β*(SE) = − 56.5(15.2), *p* < .001) and E1G (*β*(SE) = − 722.6(247.6), *p* = .004), resulting in a non-significant relationship with the T/E1G ratio (*β*(SE) = − 0.00(0.0), *p* = .110). BMI was found to interact with number of months since LMP in predicting testosterone levels (*p* = .001) such that a strong positive relationship between testosterone and months since LMP was evident among healthy weight women (*β*(SE) = 916.1 (172.2), *p* < .0001) but not among overweight (*p* = .132) or obese (*p* = .429) women (Fig. 2). Similarly, a significant interaction between BMI and months since LMP was found for the T/E1G ratio (*p* = .001) such that a positive relationship between months since LMP and the T/E1G ratio was evident among healthy weight women (*β*(SE) = 0.04 (0.01), *p* < .0001) but not among overweight (*p* = .625) or obese (*p* = .159) women (Fig. 3).

Smoking status was also significantly associated with testosterone levels such that testosterone was higher among both past smokers (*β*(SE) = 596.2(182.2), *p* = .001) and current smokers (*β*(SE) = 1314.3(551.8), *p* = .018) compared to never smokers. However, smoking status did not predict E1G levels or the T/E1G ratio (*p*s > .05) nor did it interact with months since LMP to predict hormone levels (*p*s > .05). No other baseline variables listed in Table 1 were found to interact with months since LMP in predicting testosterone or E1G levels.

### The relationship between T, E1G, and the T/E1G ratio and perimenopausal symptoms

#### Average hormone effects

**Fig. 4:**
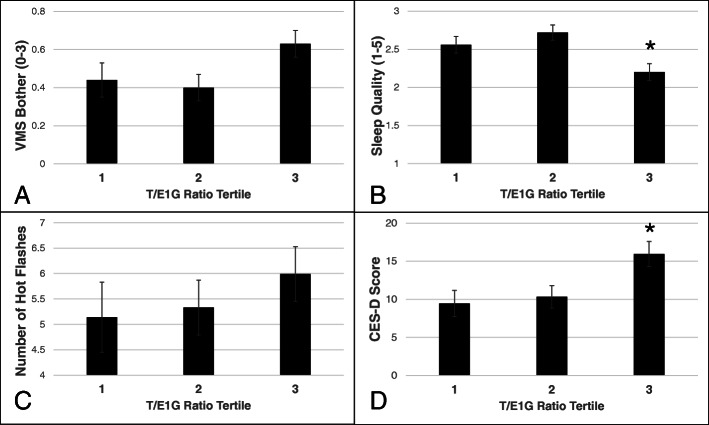
Perimenopausal symptoms by mean T/E1G tertile, statistically adjusting for mean T and mean E1G levels. *p < .05 relative to the other two tertiles

As shown in Table 2, higher average testosterone levels and a higher average T/E1G ratio were associated with more VMS bother. The effect was relatively small, however, with 1 standard deviation increase in testosterone being associated with a 0.1-point increase in VMS bother (on a scale from 0 to 3). A higher mean T/E1G ratio was also associated with poorer sleep quality, independent of both testosterone and E1G levels, such that a one standard deviation increase in the T/E1G ratio was associated with a 0.34-point drop in sleep quality (on a scale from 1 to 5). Figure 4 depicts VMS bother, number of VMS, sleep quality, and depressive symptoms by mean T/E1G, split into tertiles. It should be noted that although the continuous relationship between mean T/E1G on CES-D score was not significant, the relationship between mean T/E1G tertile on CES-D did reach statistical significance (*p* < .05) such that women in the highest T/E1G tertile endorsed more depressive symptoms than those in the other two tertiles (*p*s < .05). Unsurprisingly, mean E1G levels were inversely related to both VMS bother and frequency.

**Table 2:** Mean and weekly effects of hormones on mood and somatic symptoms. Estimates reflect the magnitude of change in the dependent variable associated with 1 SD change in the independent variable

	Testosterone		E1G		T/E1G, adjusting for T and E1G	
	Estimate (SE)	***p***-value	Estimate (SE)	***p***-value	Estimate (SE)	***p***-value
**Mean hormone levels**						
VMS number	0.22 (0.38)	.568	− 1.38 (0.59)	**.011**	0.62 (0.48)	.198
VMS bother	0.19 (0.09)	**.036**	− 0.20 (0.09)	**.043**	0.14 (0.06)	**.018**
Sleep quality	− 0.04 (0.09)	.587	0.10 (0.06)	.136	− 0.34 (0.09)	**.0001**
CES-D score	− 1.12 (0.85)	.502	− 2.07 (1.76)	.243	1.65 (1.39)	.241
**Week-to-week hormone levels**						
VMS number	0.05 (0.24)	.833	− 0.25 (0.14)	**.070**	0.19 (0.20)	.345
VMS bother	0.09 (0.04)	**.049**	− 0.07 (0.00)	**.020**	0.01 (0.04)	.800
Sleep quality	0.07 (0.08)	.391	− 0.05 (0.06)	.403	− 0.07 (0.09)	.412
CES-D score	− 0.16 (0.82)	.841	− 0.34 (0.48)	.377	1.57 (0.76)	**.041**

#### Week-to-week hormone effects

**Fig. 5:**
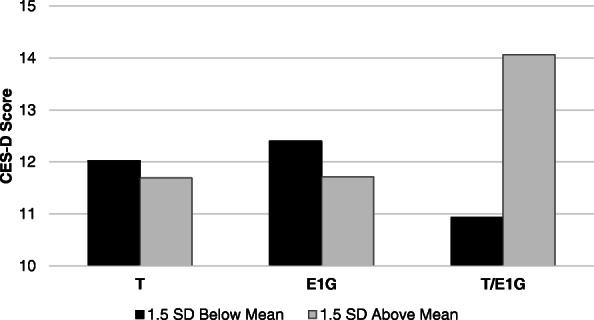
Model-based estimates of the week-to-week effects of testosterone, E1G, and the ratio between testosterone to E1G (T/E1G) on CES-D score. *p < .05

Week-to-week testosterone levels were positively associated with VMS bother while week-to-week E1G was inversely related to VMS outcomes (Table 2). A higher weekly T/E1G ratio was associated with greater depressive symptoms (Table 2; Fig. 5).

## Discussion

The current study observed a significant positive relationship between months since LMP and levels of testosterone, suggesting that, on average, testosterone levels rise as women approach postmenopausal status. In conjunction with the rise in testosterone was an expected decline in E1G, resulting in an increase in the T/E1G ratio over time. However, several baseline characteristics were found to influence hormone levels. Obesity was associated with a blunted relationship between testosterone and months since LMP, a blunted relationship between LMP and the T/E1G ratio, as well as lower testosterone levels overall. Smoking was also associated with higher testosterone levels, regardless of months since LMP. Furthermore, greater androgenicity, either indicated by testosterone levels or the T/E1G ratio, was associated with more frequent vasomotor symptoms, greater sleep disruption, and more depressive symptoms.

The rise in testosterone with advancing reproductive age observed in the current study is consistent with the Penn Ovarian Aging Study (POAS), which recruited premenopausal women and observed a rise in total serum testosterone across 14 annual assessments [5]. In contradiction to these findings, the Malmo Perimenopausal Project found that serum testosterone levels measured every 6 months prior to the onset of menopause demonstrated a small decline over time [9], and the Study of Women Across the Nation (SWAN) found that testosterone levels in annual serum samples were unchanged across the menopause transition [4, 8]. It is noteworthy that the current study measured perimenopausal testosterone levels with greater frequency than these previous studies; thus, it is possible that a brief rise in testosterone occurs in the months prior to reaching postmenopausal status but that this rise has not been adequately captured in studies using annual or semi-annual sampling. A transient increase in testosterone in the menopause transition would be consistent with the fact that the menopause transition has been associated with extreme elevations in estradiol [3] and our observation that testosterone and estradiol levels are positively correlated.

The finding that obesity was associated with lower testosterone levels is consistent with some prior results and inconsistent with others. In the Malmo Perimenopausal Project, BMI was negatively correlated with LH and FSH, suggesting an inhibitory effect of obesity on the reproductive axis [9], though this study did not observe a direct relationship between BMI and testosterone. Testosterone has also been found to be inversely associated with BMI in men, again hypothesized to result from obesity’s inhibitory effect on gonadotropin release [29]. However, in contrast to our findings, the SWAN study found a positive (albeit non-significant) relationship between BMI and testosterone [8], and in general, studies of non-perimenopausal women have found that BMI is positively correlated with testosterone levels [30–32], which may be due to testosterone’s influence on muscle mass and bone mineral density [33]. Evidently, more research is needed to tease apart the relationship between BMI and testosterone while taking into account reproductive stage and sex. The finding that past and current smoking were associated with higher testosterone levels is consistent with research conducted in reproductive-aged [31] as well as postmenopausal women [34].

In our study, vasomotor symptom bother was associated with greater androgenicity, as indicated by mean and week-to-week testosterone, as well as mean T/E1G ratio. These findings are consistent with an earlier study of perimenopausal women in which a higher ratio of testosterone to estradiol was positively associated with the frequency of vasomotor symptoms [11]. It is unknown how the interactive effects of increasing testosterone and decreasing estradiol levels may affect vasomotor symptoms in perimenopausal women. Although decreasing estradiol levels are hypothesized to influence vasomotor symptoms by modulation of neurotransmitters and temperature control in relevant brain areas [35], little is known about the role of testosterone in VMS, or how it might interact with estradiol to influence VMS.

Although we report no significant association between mean or week-to-week levels of testosterone and estradiol on CES-D score, we observed a positive relationship between week-to-week T/E1G ratio and CES-D score. Women exhibiting a mean T/E1G ratio in the top tertile were also found to have a higher CES-D score relative to women in the bottom two tertiles. This finding suggests that the relative androgenicity of the hormonal environment may be a more important predictor of perimenopausal mood than absolute levels of estradiol or testosterone. Other reports such as the SWAN study and the Penn Ovarian Aging Study (POAS) have observed a positive association between serum testosterone levels and CES-D scores [4, 5] but did not assess the ratio between testosterone and estradiol. To our knowledge, the only other study that has examined the relationship between relative androgenicity and depressive mood during the menopause transition is the study by Bromberger and colleagues [4], in which no association between relative androgenicity and depressive symptoms was found.

A number of studies have examined the effect of testosterone on postpartum mood and found levels to be associated with greater depressive symptoms. For example, one longitudinal study assessing perinatal mood and steroid hormone levels in a large sample of 193 women found that higher serum testosterone was associated with depression and anger in the first few days postpartum [36]. Albeit with a small sample size (*n* = 15), another study found higher serum testosterone to be associated with greater depressive symptomology, anger, and tension and hostility early in the postpartum period but not in the weeks before birth [37]. Given that both the postpartum period and late menopause transition are both associated with estradiol withdrawal, these findings may suggest that the negative mood effects of testosterone are amplified in the context of low or decreasing estradiol levels. This would help to explain why the T/E1G ratio was a stronger predictor of depressive mood than testosterone alone.

The mechanisms by which the ratio of testosterone to estradiol would negatively impact mood remains unclear. However, one possibility may relate to estradiol’s effects on another androgen, dehydroepiandrosterone (DHEA). Although overall findings are somewhat mixed, a number of studies have observed a beneficial mood effect of DHEA in women [38], including one of the perinatal mood studies described above [37]. Estradiol administration has been shown to decrease DHEA levels [39] — it may therefore be that periods of low estradiol result in a higher ratio of testosterone to DHEA, creating a more pronounced negative mood effect. This remains pure speculation, however — future research is needed to confirm this hypothesis and to further investigate the potential explanations for our findings.

Several limitations of the current report should be noted. First, the study was restricted to a 12-week analysis of perimenopausal women, which may underrepresent the variability of hormones across the entire menopause transition. Second, the majority of participants in our sample were late-perimenopausal both at baseline and at the end of the study (Table 1), and only a small portion (5%) transitioned to early postmenopausal status. Thus, although our sample included early- and late-perimenopausal women throughout the study and a small number of early postmenopausal women by the end of the study, our results may be more relevant to women later in the menopause transition. Third, our sample was restricted to women without a diagnosis of major depressive disorder at baseline; the results may therefore not generalize to this population. Future research examining the effect of testosterone on mood in a sample of women with a wider range of depression scores is warranted. Fourth, although our study was well powered to detect the effects of interest, its small sample size may limit the generalizability of our findings. Finally, the subjective nature of the behavioral outcomes should be considered; sleep quality, depressive symptomology, and vasomotor symptoms were all self-report measures. Despite these limitations, the FEMM study provides a unique contribution to understanding the role of hormones in the pathology of depression during the menopause transition.

## Perspectives and significance

In conclusion, the current study suggests that the ratio of testosterone to estradiol increases with advancing reproductive age in the transition to menopause and that a higher ratio is associated with worse vasomotor symptom bother, poorer sleep quality, and more depressive mood. To our knowledge, the current study is the first longitudinal study of perimenopausal women to make use of frequent and concurrent assessments of testosterone and mood. In addition, our study is one of few to investigate the role of the relative androgenicity of the hormonal environment in the etiology of perimenopausal depression. Our findings suggest that testosterone and estradiol may interact to predict perimenopausal symptoms and that the ratio between these hormones may be an important variable to calculate in the future. Further research is needed to investigate the physiological and psychological mechanisms by which these two hormones may interact to predict perimenopausal symptoms.

## Data Availability

The datasets generated and analyzed during the current study are not publicly available due to concerns of patient confidentiality but are available from the corresponding author on reasonable request.
